# Green Formulation of Menadione-Loaded Niosome as a Skin-Lightening Preparation: *In Vitro* /*In Vivo* Safety Evaluation on Wistar Rat

**DOI:** 10.34172/apb.42731

**Published:** 2024-09-15

**Authors:** Majid Saeedi, Katayoun Morteza-Semnani, Jafar Akbari, Seyyed Mobin Rahimnia, Fatemeh Ahmadi, Mohammad Reza Mojaveri, Saghar Ahmadipour, Seyyed Mohammad Hassan Hashemi

**Affiliations:** ^1^Department of Pharmaceutics, Faculty of Pharmacy, Mazandaran University of Medical Sciences, Sari, Iran.; ^2^Pharmaceutical Sciences Research Center, Hemoglobinopathy Institute, Mazandaran University of Medical Sciences, Sari, Iran.; ^3^Department of Medicinal Chemistry, Faculty of Pharmacy, Mazandaran University of Medical Sciences, Sari, Iran.; ^4^Student Research Committee, Faculty of Pharmacy, Mazandaran University of Medical Sciences, Sari, Iran.; ^5^Food Health Research Center, Hormozgan University of Medical Sciences, Bandar Abbas, Iran.; ^6^Department of Pharmaceutics, Faculty of Pharmacy, Hormozgan University of Medical Sciences, Bandar Abbas, Iran.

**Keywords:** Green technique, Anti-melanogenesis, Menadione, Niosome, Cosmetic

## Abstract

**Purpose:**

In the present research, a green technique (an ultrasonic method) was used to synthesize menadione sodium bisulfite (MSB) niosome (Menasome) which is used to improve dermal delivery and increase anti-melanogenesis activities.

**Methods:**

Various cholesterol: surfactant (Chol: Sur) ratios were investigated to optimize the Menasomes. Photon correlation spectroscopy, attenuated total reflectance-Fourier transform infrared spectroscopy (ATR-FTIR), transmission electron microscopy (TEM), and differential scanning calorimetry (DSC) were employed to characterize the solid state of MSB in nanoparticle form. Additionally, the optimized formulation was used to investigate *ex-vivo* skin absorption, *in vivo* skin irritation, *in vitro* cell survival, and anti-melanogenesis activity.

**Results:**

The results exhibited that increasing cholesterol declined the average size of the Menasomes from 653.766±25.171 nm to 298.133±8.823 nm and increased entrapment efficiency 30.237±3.4204% to 83.616±2.550 %. The rat skin permeation study indicated that Menasome gel administered more MSB in dermal layers (439.000±36.190 μg/cm^2^ or 23.827±1.964%) than MSB plain gel (286.200±22.6 μg/cm^2^ or 15.53±1.227%). In both the *in vivo* skin irritation test and the *in vitro* cytotoxicity experiment, the extended-release behavior of the enhanced Menasome demonstrated a minimal side effect profile. Furthermore, optimum Menasome inhibited melanin formation (37.426±1.644% at 15μM) greater than free MSB (57.383±1.654%) considerably (*P*<0.05). Furthermore, Menasome 7 prevented L-dopa auto-oxidation in higher levels (95.140±2.439%) than pure MSB solution (83.953±1.629%).

**Conclusion:**

According to the study’s findings, the prepared Menasome could be employed as a viable nanovehicle for MSB dermal delivery, a promising solution for the management of human hyperpigmentation disorders.

## Introduction

 Frequently, cutaneous melanin is regarded as a widespread class of biological pigments.^1^ The complex chemical mechanism involving multiple enzymes causes the melanin synthesis stage in epidermal melanocytes.^2^ The primary enzyme involved in the synthesis of melanin is tyrosinase, also known as monophenol or o-diphenol oxygen oxidoreductase. It is an enzyme that contains copper.^3^

 The most common skin diseases caused by abnormal melanocyte distribution, function, and structure are pigmentation conditions. For most populations, especially women, the management of pigmentation problems has long been dissatisfying and difficult. A large proportion of middle-aged women frequently have aberrant facial pigmentation brought on by various endogenous and exogenous factors.^4^ Reduced skin pigmentation (hypopigmentation) and enhanced skin pigmentation (hyperpigmentation) are two kinds of pigmentation problems. These skin pigmentation disorders might have psycho-social impacts as well as cognitive, mental, and aesthetic challenges for the patients. Skin pigmentation disturbances are linked to common pigmentation diseases such as Melasma (Chloasma) and Ephelides (freckles).^5^

 Certain skin lighteners can inhibit the synthesis of melanin, which makes them potentially useful as a treatment for hyperpigmented complaints.^6^ Menadione sodium bisulfite (MSB) can prevent the production of melanin in a dose-dependent manner as a result of decreased tyrosinase activity.^7^ One of the products of menadione (vitamin K3), which has a high water solubility, is MSB.^8^

 For hydrophilic drugs, like MSB, delivery systems development is crucial, but there are several problems in the way of hydrophilic and macromolecule drug delivery.^2^ These obstacles include (*a*) unsuitable and low permeability skin penetration due to hydrophilic structure, (*b*) unsuitable half-life in the biological fluid, and (*c*) low bioavailability and hydrolytic degradation.^9^ To overcome this challenge, researchers have developed nano-formulations that encapsulate hydrophilic drugs.

 Higher dosages of drugs could be successfully delivered through the skin using lipidic nanocarriers, which could release drugs at controlled rates.^10^ According to Manconi et al, niosomes are generally non-toxic and safe, have low production prices, and have sufficient stability over a prolonged duration. In 1975, niosomes were discovered and advanced for topical usage and after that, numerous fabrication industries have paid much attention to the technology of niosomes, containing the anti-aging Lancome product Niosome Plus.^11^ According to Manconi et al, the skin depot reached via a liposomal platform yielded the lowest dermal concentration, whereas the niosomal formulation of tretinoin, manufactured utilizing the thin film hydration method, produced the highest dermal concentration.^12^ In other research, To improve arbutin’s cutaneous permeability, Span 60, a non-ionic surfactant, was used to create arbutin niosomes through thin film hydration.^13^ To increase arbutin’s skin permeability, arbutin niosome (Arbusome) was made in a different study utilizing an ultrasonic technique and non-ionic surfactants (Tween and Span 20).^14^ In a previous study, it was discovered that the epidermal absorption capacity of ellagic acid niosomes produced by reverse phase evaporation was higher than that of ellagic acid solutions.^15^ To increase the skin’s ability to absorb kojic acid, other research has produced different types of nanoparticles using alternate methods, for example, nanostructured lipid carriers and solid lipid nanoparticles.^2^

 In comparison with other approaches (reverse phase evaporation and thin film hydration), the ultrasonic technique (as a green technique) has the advantage of not requiring the usage of an organic solvent.^16^ Furthermore, the background of alternative methods and the ultrasonic technique differ when considering the preparation of niosomes. The ultrasonic technique, also known as the high energy technique, creates niosomal structures by applying a high system energy input for a short period. High cavitation forces are produced by the ultrasonic approach, which effectively mixes the system.^17^ However, changes occur much more slowly during the multistep process of thin film hydration and reverse phase evaporation method.^18^

 As far as we are aware, no study has been done to assess the MSB niosomal formulation employing an economical and environmentally friendly technique like ultrasonication as a carrier for topical administration. Therefore, as an eco-friendly and green alternative to conventional carriers for topical distribution, MSB-encapsulated niosomes (Menasome) were produced as cosmeceutical products (Menasome gel) utilizing ultrasonication. In order to achieve optimum skin penetration, the niosomal formulation can also be improved by changing the cholesterol to surfactant (Span 20 and Tween 20) ratio (Chol:Sur ratio). Furthermore, absence of investigation on the inhibitory impression of MSB noisome on the procedure of L-DOPA auto-oxidation and the quantification of melanin in the B16F10 cell line. Also, there is an absence of examination of cutaneous sensitivity produced by MSB-niosome on Wistar rats.

## Materials and Methods

###  Materials

 In this study, Tween 20 (Sharlua, Sharlab S.L., Spain), Span 20 (Samchun Pure Chemical Co., Ltd. Korea), Menadione sodium bisulfite (Sigma, Germany), and Cholesterol (Merck, Germany) were applied. Carbopol 941 was obtained from B⋅F Goodrich, USA.

###  Preparation of Menasome

 An ultrasonic procedure was done to prepare the Menasome.^14,16^ Tween 20, Span 20, and Chol were combined in a crystal beaker using magnetic stirring at 70 °C (organic phase). In the aqueous medium, the temperature of MSB (1%)^19^ and water was raised to equal levels with the organic phase. A magnetic stirrer set to 600 rpm for 30 minutes with a hot plate was used to mix the two processes to create a pre-niosome. To create Menasome, the mixture was then rapidly frozen in an ice reservoir bath after being sonicated with a probing sonicator (Table 1). The RHLB value can be calculated using the following formula:


(1) $$RHLB=f\times HLB\;\left(Tween\;20\right)\;+\;\left(1-f\right)\;HLB\;\left(Span20\right)$$

Which f is a fraction of surfactant in a mixture of surfactants.

**Table 1:** Component and physicochemical properties of investigated Menasome (Mean ± SD, n = 3)

**Formulation**	**MSB (%)**	**Chol (%)**	**Span 20 (%)**	**Tween 20 (%)**	**Chol/Sur ratio **	**Water up to ml**	**Particle size** **nm**	**PDI **	**Zeta potential (mv)**	**EE (%)**
Menasome 1	1	0	0.75	0.75	0:6	20	653.766 ± 25.171	0.536 ± 0.0136	-8.156 ± 0.285	30.237 ± 3.420
Menasome 2	1	0.25	0.75	0.75	1:6	20	516.300 ± 12.522	0.513 ± 0.006	-7.540 ± 0.450	38.678 ± 2.977
Menasome 3	1	0.5	0.75	0.75	2:6	20	470.600 ± 15.070	0.493 ± 0.010	-6.513 ± 0.441	45.958 ± 2.510
Menasome 4	1	0.75	0.75	0.75	3:6	20	427.766 ± 13.136	0.4577 ± 0.018	-5.740 ± 0.557	43.477 ± 2.537
Menasome 5	1	1	0.75	0.75	4:6	20	393.233 ± 15.157	0.392 ± 0.018	-5.520 ± 0.501	65.420 ± 3.300
Menasome 6	1	1.25	0.75	0.75	5:6	20	332.300 ± 10.068	0.357 ± 0.013	-4.746 ± 0.363	73.3018 ± 1.504
Menasome 7	1	1.5	0.75	0.75	6:6	20	298.133 ± 8.823	0.3047 ± 0.006	-3.056 ± 0.797	83.616 ± 2.5508

Abbreviations: Chol: Sur, cholesterol: surfactant; MSB, menadione sodium bisulfite; PDI, polydispersity index; EE, Entrapment efficiency.

###  Menasome characterization 

 The average size of Menasome preparations and polydispersity index (PDI) was achieved at 25 °C using a Zetasizer Nano ZS system (Malvern Instruments Worcestershire, UK) and a dynamic light scattering (DLS) Mastersizer 2000 (Malvern Panalytical Technologies, UK). Laser Doppler electrophoresis was used to conclude the zeta potential of Menasomes.^20^

###  Formulating of Menasome gel and MSB simple gel 

 To distribute 1.5% w/v of Carbopol 941, 1% w/w of Menasome was combined with 0.1% sodium benzoate-filtered preserved water, and the mixture was stored for 24 hours. The combination of carbopol in the Menasome dispersion was neutralized using 100 mg triethanolamine applying a propeller homogenizer that rotates at 400 rpm. MSB solution was combined using a propeller homogenizer operating at 400 rpm (1% w/w MSB) with 1.5% w/v Carbopol 941 to form an MSB simple gel.

###  Entrapment efficiency (EE %)

 The prepared Menasomes were centrifuged (SIGMA; 3-30 KS: Germany) for 45 minutes at 18 000 rpm and then filtered with 0.22 m pore size to determine the EE%. After that, the amount of MSB in the filtrate (free drug) was measured using a UV spectrophotometer: JASCO V-630 (UV ± Vis, UK) at 264 nm.


(2) $$EE\%=((W_{initial}-W_{free})/W_{initial})$$

Where Wfree indicates the total amount of material in the supernatant and Winitial indicates the amount of material added to the formulation.

###  Microscopic investigation

 The morphology of the Menasome was examined using transition electron microscopy. After applying a gold coating, the sample was analyzed using a HITACHI S-4160 SEM (Japan) with a magnification of 15,000 and an acceleration voltage of 20.0 kV.

###  Differential scanning calorimetry (DSC) Assessment

 DSC was used to examine the parts and Menasome powder (Pyris 6, PerkinElmer; USA). To create Menasome powder, the niosomes were separated from the dispersion using a centrifuge (3–30 ks Sigma centrifuge). The alpha 1-2 LDplus freeze drier was utilized to freeze-dry the separated niosomes at a lower pressure (Marin Christ, Osterode, Germany). MSB, cholesterol, and freeze-dried Menasome specimens totaling around 5 mg were quantified and placed in aluminum pans. For thirty minutes, the covered pans were kept in an isothermal atmosphere at a temperature of 20 °C. Following equilibrium, the physical mixture, Menasome powder, and original drug components were subjected to a temperature of 25-300 °C at a heating rate of 20 °C/min in an environment of inert N_2_ gas. The DSC spectra of these samples were then recorded. The DSC was calibrated with indium.^20^

###  Attenuated total reflectance-Fourier transform infrared spectroscopy (ATR-FTIR) Spectroscopy 

 To examine the MSB–excipient interaction, a diamond ATR-equipped FTIR spectrophotometer Cary 630 (Agilent Technologies Inc., CA, USA) was used. MSB, Span 20, Tween 20, cholesterol, and Menasome (freeze-dried powder) were done for the ATR-FTIR analyses. The observed spectra ranged between 4000-400 cm^-1^.^21^

###  Drug release test

 Immersion cells with a cellulose acetate membrane (MWCO of 12 kDa) were used for the *in vitro* release study. The samples were positioned into the cells, and then the caps were placed on and an acetate cellulose membrane was placed over the cells. Within the USP Dissolution Apparatus II, the cells were installed.^22,23^ A dissolution medium volume of around 900 mL was produced for the release test at a speed of 100 rpm and 37.0 °C. At various time intervals (2, 4, 6, 8, and 24 hours), the dissolving medium (5 mL) was extracted and filtered using 0.22 micrometer filter paper. A UV light with a wavelength of 264 nm was used to determine the drug content of the samples. Following each sampling interval, pH 6.8 buffer phosphate (5 mL) was added to maintain a constant volume of the dissolving solution.

###  Ex-vivo dermal absorption investigations

 The UK Animals (Scientific Procedures) Act 1986 and its related guidelines, the ARRIVE regulations, and EU Directive 2010/63/EU on animal research were all followed during the animal investigations. A mixture of 13 mg xylazine/kg 87 mg and ketamine/kg body weight was utilized to anesthetize male Wistar rats weighing 120-150 g, and electric razors were utilized to shave their abdomen skin. Following a 48-hour time, the rats were put to sleep via breathing in chloroform, and surgically removed the skin of the abdomen. Before the diffusion investigations started, all subcutaneous lipids were cautiously eliminated, and for 24 hours at 4 °C, the skin was immersed in a saline solution. After that, the skin was put in Franz cells with a 3.8 cm^2^ diffusional area to evaluate permeation. The removed skin was positioned with its dermis exposing the recipient fluids, situated between the donor and recipient compartments. After that, purified double-distilled water (pH 5.5 equivalent to normal skin pH) was placed within the diffusion cells, maintained at 32 ± 0.5 °C during the tests.^24,25^ Since the aim of this test is to examine the amount of medicine passing through the skin, the receptor should have the ability to dissolve the medicine completely. In other words, it should be able to maintain the sink condition according to USP pharmacopeia 43. For this reason, purified double-distilled water (pH 5.5) was chosen for the dissolution medium. The cell chamber was surrounded by a jacket that enabled water to circulate, and magnetic stirring rods were used to agitate the mixture at 150 rpm. 1 g of Menasome gel preparations including MSB (10 mg) and 1 g of simple MSB gel combinations, which contained the same value of MSB as the niosome formulations, were equally administered to the skin’s trimmed dorsal portions in the donor chamber that were sealed off from the environment. At specific intervals (2, 4, 6, 8, 10, and 24 hours), the sample (5 mL) was taken out of the receiver medium and substituted with an equivalent amount of fresh water. A UV spectrophotometer with a 268 nm setting was applied to determine the amount of drug in the removed samples.

###  Skin MSB retention examinations

 As previously stated, the skins were removed following the last stage of the permeation studies. Before determining the amounts of MSB deposited in the skin, they were washed with water 3 times followed by drying. Using scissors, the skin was sliced into tiny pieces. After being put into tubes, it was dissolved in water and subjected to an hour of sonication using a bath sonicator. The final solution was filtered using filter paper and a 0.22 µm syringe filter, and a UV spectrophotometer was used to quantify the concentration of the mixture at 264 nm.

###  Cell survival research 

 Human foreskin fibroblast (HFF) cells from the National Cell Bank (Pasteur Institute of Iran) were used to test the preparations’ cell survival *in vitro*. Following that, a mass of 10^5^ cells were placed onto the Nunclon microplate base for a duration of 24 hours with different concentrations of MSB, niosome without medication, and Menasome (15, 10, 7.5, 5, 2.5, and 1 µM) or the carrier control. After the materials were removed, the cells were cleaned with PBS, and colorimetry formazan (MTT) was utilized to evaluate the cells’ viability.^26^ The cells were then incubated for four hours at 37 °C after MTT (0.5 mg/mL) was added. Furthermore, after discarding the supernatant, the silt was dissolved in dimethyl sulfoxide (DMSO) (100µL) comprising formazan crystals. Following a 20-minute shake of the plates, the optical density at 560 nm was measured by applying a multi-walled spectrophotometer. Six more controls (the cells in the medium) were examined three times at various doses (15 10, 7.5, 5, 2.5, and 1 µM) to evaluate the cell’s viability. The vitality of the cells was measured as follows:


(3) $$\%Cell\;survival=\left(Abs\;Sample-Abs\;Blank\right)/\left(Abs\;Control-Abs\;Blank\right)$$

###  L-DOPA auto-oxidation inhibitory test

 Much of the previously stated calculation was used to determine the formulation and pure drug’s preventive effect on L-DOPA auto-oxidation. To summarize, sodium phosphate buffer (0.1 M at pH 6.5) was prepared in 96-well plates, and different quantities of MSB and Menasome were prepared. A 96-well plate was filled with 300 μL of 10 mM L-DOPA and sodium phosphate buffer (0.1 M at pH 6.5). The blend was then incubated for 15 minutes at 37 °C. By measuring absorbance at 475 nm, activity was determined.^19^

###  Calculation of melanin amount of melanoma cells

 Murine B16F10 melanoma cells were achieved at the Pasteur Institute.^27^ Murine B16F10 melanoma cells were seeded in complete RPMI media supplemented (streptomycin (0.1 mg /mL), penicillin (100 units /mL), 10% fetal bovine serum (FBS; Gibco, USA)) in a 12-well plate overnight. Following a 24-hour incubation period, the cells were treated with MSB and Menasome. For thirty minutes at 100 °C, the cell pellets were dissolved in a 100 μL solution of NaOH (2 M). By measuring their absorptions at 405 nm, the melanin content of the samples was compared to the control group via a Microplate Reader (BioTek, Winooski, USA).

###  In vivo skin irritation investigations

 The Institute Animal Ethical Board criteria were adhered to throughout the investigations. Wistar rats were shaved the day before the experiment to remove any hair from their dorsal regions. Group V was given a 0.8% (v/v) water solution of formalin as the standard irritant, Group I was the control, and Group IV received the free drug niosome. Menasome gel and MSB simple gel were given to groups II and III. For three days, Menasome gel, MSB simple gel, formalin solution, and a placebo were applied topically to the skin of rats. The affected area was examined visually throughout the time.^19,28^ This procedure is blinded for observers. After 72 hours, the sites of application were examined for edema and erythema and graded on a scale from zero to 4 based on visual assessment by the same blind investigator. The grading scale was as follows: 0 for none, 1 for slight, 2 for well-defined, 3 for moderate, and 4 indicating scar or ulcer formation. Edema was graded similarly: 0 for none, 1 for slight, 2 for well-defined, 3 for moderate, and 4 for severe. Photographs were taken to document erythema observed at some application sites on rats. The primary irritancy index for each group was determined using a specific calculation formula.^29^


(4) Primary irritancy index=Mean score of erythema+Mean score of edema

The formulation was classified as non-irritant if (mean score < 2)

###  Statistical evaluation 

 The mean standard deviation was used to report the study’s findings. GraphPad Prism 8 was utilized to examine the outcome. Statistical analysis was performed on the selected parameters using analysis of variance and the Tukey test LSD’s post hoc test. It was discovered that the *P* < 0.05 has statistical significance (n = 3).

## Results and Discussion

###  Characterization of Menasome

 Niosomes composed of MSB were synthesized via ultrasonication. This technique used various ratios of Chol to a binary mixture of Tween 20 and Span 20, which is a nonionic surfactant (Chol:Sur 0:6, 1:6, 2:6, 3:6, 4:6, 5:6, 6:6 w/w). Table 1 lists the characteristics of the Menasome compositions. From 0:6 (Menasome 1) to 6:6 (Menasome 7), the Menasome’s particle size decreased significantly from 653.766 ± 25.171 to 298.133 ± 8.823 nm (*P* < 0.05). It has been demonstrated that the presence of Chol enhances Menasome bilayer cohesion and rigidity.^30^ Niosomes diminished in size as a result of this. Niosomes with more Chol may have a reduced radius, according to findings by Chaw and Kim^31^ and Akbari et al.^32^ Additionally, the probe’s increased energy output may have contributed to the excessive heat generation that resulted in the decreased particle size. Furthermore, the characteristics of the MSB vesicles can be adjusted to reach an optimal value in terms of size by utilizing the dual combinations of surfactants (Tween 20 and Span 20), which control the amount of HLB.^33^ Table 1 also displays each Menasome encapsulation efficiency (EE percent). Table 1 displays the range of percentages of MSB entrapped in Menasomes from 30.237 ± 3.420 to 83.616 ± 2.550%. When the ratio of Chol to sur was raised to 6:6, the EE % increased due to the rise in bilayer rigidity and a decrease in drug leakage from the niosome membrane. It has been demonstrated that Chol influences EE % and membrane permeability, leading to less permeable niosomes.^34^ Additionally, surfactants with low (Span 20 HLB value 8.6) and high (Tween 20 HLB 16.7 value HLB values could be dispersed in the oily and aqueous phases, respectively, therefore enhancing the physical stability at the interface of the surfactant films. Also, for obtaining stable niosome and successful emulsified Chol, the ratio of surfactant (Span 20 and Tween 20) was considered constant (0.75%) to each other as mentioned in previous studies.^14,32^ In order to create a stable emulsion in water, it is important to have an HLB value above 10 (in this case 12.65) because this indicates that the HLB is more hydrophilic than lipophilic. Emulsifiers (Tween 20 with HLB 16.7) with an HLB value above 10 effectively stabilize oil-in-water emulsions, where water is the continuous phase and oil is the dispersed phase. These emulsifiers help to reduce the surface tension between the oil and water, thereby preventing the oil droplets from coalescing and ensuring a stable emulsion. In oil-in-water emulsions, using a hydrophilic emulsifier beyond a certain point becomes ineffective for high oil concentrations because these emulsifiers are primarily water-soluble. As the oil content increases, the hydrophilic emulsifier cannot sufficiently stabilize the larger oil droplets, as it tends to dissolve in the water phase. This imbalance leads to an inadequate reduction in the surface tension between oil and water, causing the emulsion to become unstable. Instead, a balance between hydrophilic and lipophilic properties often represented by an appropriate HLB value, is necessary to effectively stabilize the emulsion with higher oil content (in this case the required HLB is 12.65).^35^

 Additionally, Chol forms associations with the surfactant molecules, which alter their vesicular structures and physical properties through the control of bilayer cohesion and strength, ultimately leading to an increase in EE%.^34^ Chol decreases the vesicles’ gel-liquid transition temperature, making them less permeable to drug leakage and more stable, which can raise the EE%.^36^ Mokhtar et al looked at how formulation factors, such as the amount of Chol, affected the entrapment of flurbiprofen-loaded niosomes.^30,37^ They discovered that when the ratio of Chol:Sur increased, the EE percent improved.^37^

Table 1 shows that the higher concentrations of Chol decreased the surface charge of Menasome. For instance, the negative zeta potential decreased (-3.056 ± 0.797 mV) when the ratio of Chol:Sur was increased to 6:6, but it increased (-8.156 ± 0.285 mV) when the concentration of Chol was lower. Because of the electrostatic stabilization of the niosome, Menasome 1 with a maximum zeta potential of -8.156 ± 0.285 mV may be desirable since it offers high stability to the Menasome during storage and when combined with water. The distribution of free MSB in the aqueous state or the diffusion layer potential may be what causes the finding that the percentage of MSB entrapment reduced as the zeta potential of particles increased. These results are consistent with other studies.^32,38^ It was also noteworthy that the colloidal particles were surrounded by a negative zeta potential due to the usage of nonionic surfactants. This could be explained by the nonionic surfactants’ ethoxy groups’ dipole characteristics.^39^

 According to Taymouri and Varshosaz, the PDI value normally ranged from 0 to 1, with values close to zero indicating a uniform and homogenous dispersal.^40^ The PDI value ranged from 0.304 ± 0.006 to 0.536 ± 0.013 in Table 1. A wide distribution of particle size is usually indicated by PDI values greater than 0.7.^41^ Table 1 makes it clear that formulations with 1.5% of Chol had the lowest PDI value (0.304 ± 0.006). Conversely, Menasome 1 with the lowest level of Chol—showed the highest PDI value (0.536 ± 0.013). In addition to Chol concentration, other factors that affect the reduction in PDI value are surfactant type, surfactant HLB value, sonication power, and particle charge.^18,21^ Additionally, the Chol regularly decreases the radius and number of the bilayers as well as the chain order of gel state bilayers.^42^ Ultimately, the normalized distribution gets smaller due to the reduction in relation to the radius or number of bilayers, as per the thermodynamic scaling effect for soft self-assembling particles.^43^

 The absence of organic solvent requirements in the current niosome manufacturing process makes it a more desirable approach compared to other methods. The physical experience of traditional (such as thin-film hydration) and Ultrasonic methods is quite diverse when considering the construction of niosomes. The high-energy method known as the ultrasonic processor methodology produces niosomal structures by supplying a large amount of energy to the system during a short period. Higher cavitation forces produced by the ultrasonic processing method efficiently mix the material and produce smaller niosomes than the traditional approach. However, changes occur much more slowly throughout the multistep process of thin-film hydration. Niosomes are often made using traditional thin-film techniques, which frequently call for the addition of organic solvents at one or more stages throughout the procedure. Human health is at risk of solvents from these processes.^18^ Moreover, none of the studies examined the impact of the Chol: Sur w/w percent ratio—which is necessary to increase the efficiency of Menasomes. Particle size, PDI, zeta potential, and drug entrapment percentage of the ultrasonicated optimal Menasome 7 are 298.133 ± 8.823 nm, 0.304 ± 0.006, -3.056 ± 0.797mV, and 83.616 ± 2.550%, respectively.

###  ATR-FTIR analysis

**Figure 1:**
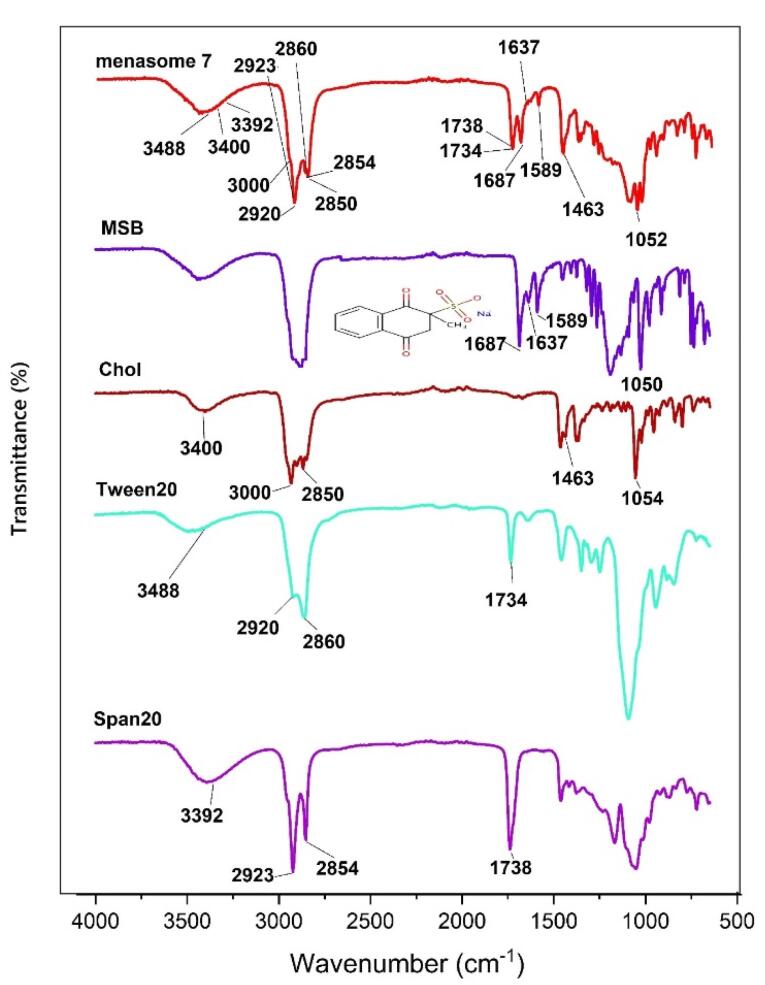
ATR-FTIR spectra of Menasome 7, MSB (menadione sodium bisulfite), Chol (cholesterol), Tween 20, and Span 20

 The chemical interaction between the drug and the excipients utilized to make the niosome was examined in ATR-FTIR spectroscopy. The ATR-FTIR spectra of MSB in pure form, Span 20, Tween 20, Chol, and Menasome 7 are shown in Figure 1. Specific peaks of MSB were detected at 1687 cm^-1^ (C = O, stretching), 1637 cm^-1^ (C = O, stretching), 1589 cm^-1^ (C-C, stretching), and 1050 (S = O, stretching). Chol showed peaks at 1054 cm^-1^ (C–O stretching), 1463 cm^-1^ (C–H bending), 3000-2850 cm^-1^ (C–H stretching), and 3400 cm^-1^ (O–H stretching). Span 20 displays diagnostic peaks at 1738 cm^-1^ (C = O stretching), 2854 cm^-1^ (–CH– symmetric stretching) 2923 cm^-1^ (–CH– asymmetric stretching), and 3392 cm^-1^ (O–H stretching); and Tween 20 presented peaks at 1734 cm^-1^ (C = O stretching), 2860 cm^-1^ (–CH– symmetric stretching) 2920 cm^-1^ (–CH– asymmetric stretching), and 3488 cm^-1^ (O–H stretching). Figure 1 demonstrated that the ATR-FTIR spectra of Menasome 7 display specific peaks for MSB (1687 cm^-1^ (C = O, stretching), 1637 cm^-1^ (C = O, stretching) (is not considered as a distinctive peak), and 1589 cm^-1^ (C-C, stretching). Any chemical interactions between the excipient and the MSB were ruled out, as the ATR-FTIR showed.

###  DSC analysis

**Figure 2:**
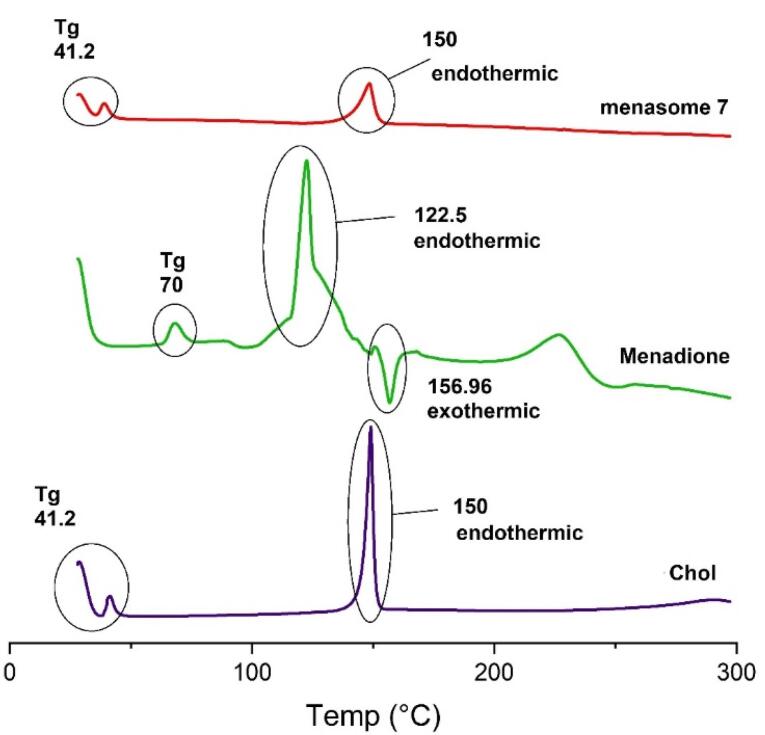
DSC traces of Menasome 7, MSB (menadione sodium bisulfite), and Chol (cholesterol)

**Figure 3:**
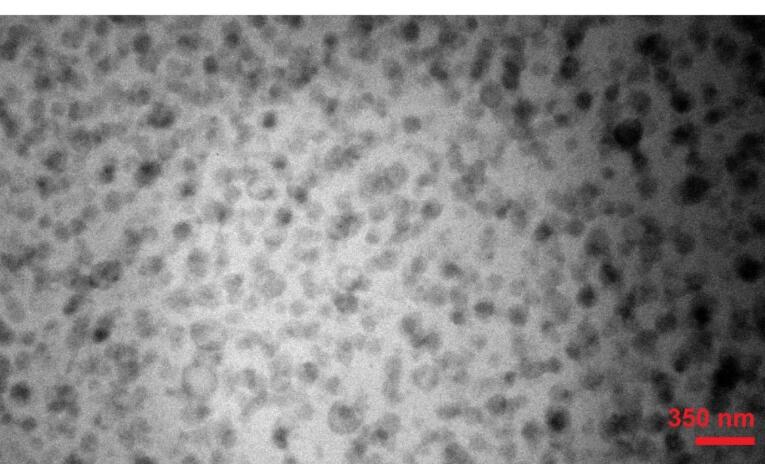
TEM image of Menasome 7 (optimum formulation)

 The DSC traces of pure MSB, Chol, and Menasome 7 are displayed in Figure 2. Thermogravimetric analysis using Chol and MSB indicated a single, strong endothermic peak at 150 °C, 122.5 °C, and exothermic 156.96 °C, respectively. These peaks showed their highly crystalline structure, fitting their melting temperatures. Also, glass transition temperature (Tg) Chol (41 °C), MSB (70 °C), and Menasome 7 (41 °C) were shown in Figure 3.

 MSB in the niosome is either molecularly dispersed within the niosome or in an amorphous state, according to the DSC thermograms of the niosome formulation under examination, which only showed the endothermic peak around the Chol melting point (Figure 2).

###  Transmission electron microscopy (TEM) analysis

 These niosomes were spherical and distinct from each other, as demonstrated by the Menasome 7 TEM image (Figure 3). In addition, the micrograph showed that the Menasome’s size is properly uniform, which is to be expected considering their morphological characteristics.

###  Drug release study

 The release of a drug from a nanoparticle gel formulation is often studied over extended periods, such as 24 hours or more, to fully understand the release profile and ensure a comprehensive evaluation of the formulation’s performance. Also, Regulatory agencies often require extended-release studies to ensure that the drug delivery system meets safety and efficacy standards over the intended duration of use, and these studies are also part of the quality control process to ensure consistency and reliability.^44^

**Figure 4:**
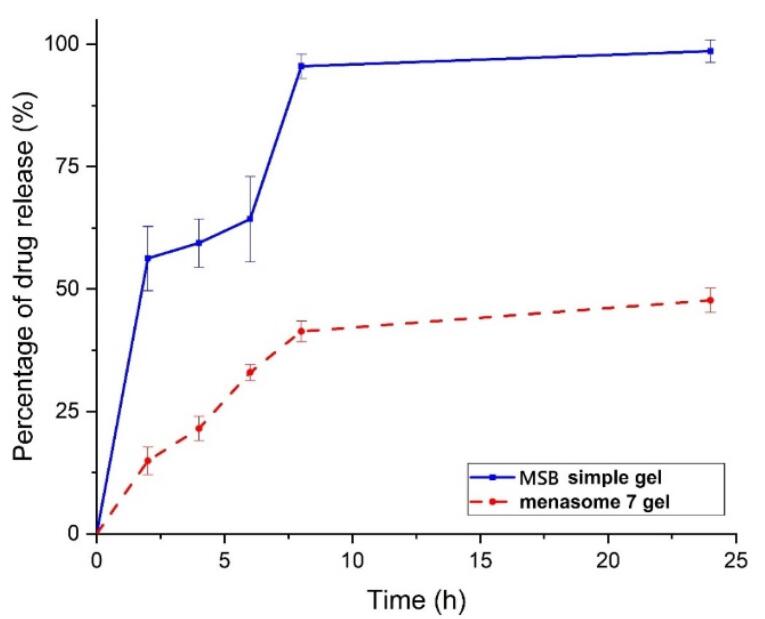
Profile of drug release from MSB (menadione sodium bisulfite) simple gel and Menasome 7 gel (mean ± SD, n = 3)

 Adjusting the drug release from the niosome has been revealed to affect drug bioavailability.^1^ The impact of simple gel and niosome formulation gel on the MSB release profile is depicted in Figure 4. The results indicated that the Menasome 7 gel released 14.920 ± 2.789% of the drug for up to 2 hours, and thereafter it displayed a continuous release profile for up to 24 hours. This was not the case for the MSB-containing simple gel, as over 94% of the drug was released in the first 8 hours.

 The release profile of a hydrophilic drug from niosomes typically follows a biphasic pattern, which includes an initial burst release (release of drug after 8 hours is 41%) followed by a slower, sustained release (47% drug release after 24 hours). Extending the drug release study beyond 25 hours would allow for a comprehensive determination of the fate of the remaining drug in the niosome and gel matrix, potentially resulting in the complete release of the drug. It has been demonstrated that Chol can reduce drug mobility in niosomes relative to the use of simple gel, enabling a more extended and sustained drug release pattern. The drug release rate from niosomes is influenced by several factors, but the most crucial ones include molecular size, matrix viscosity, high surface area, and high diffusion coefficient. If the drug is encapsulated in the niosome’s inner core, it is delivered gradually and consistently.^16^ However, if the drug is placed onto the surface or outer shell, its release can occur extremely quickly.^45^ Many efforts have been made to build niosome systems that can improve formulation effectiveness for improved drug delivery system parameters, as controlling drug release from niosomes is crucial.^46-48^ Saeedi et al have also provided information on the drug release from niosomes.^49^

###  In vitro percutaneous absorption study

**Figure 5:**
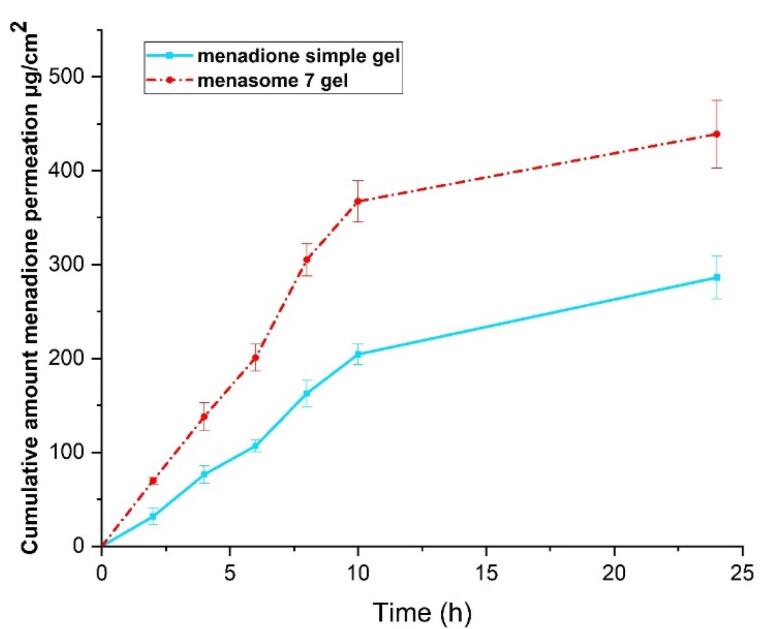
Cumulative MSB (menadione sodium bisulfite) amounts permeated across rat skin (mean ± SD, n = 3)

**Figure 6:**
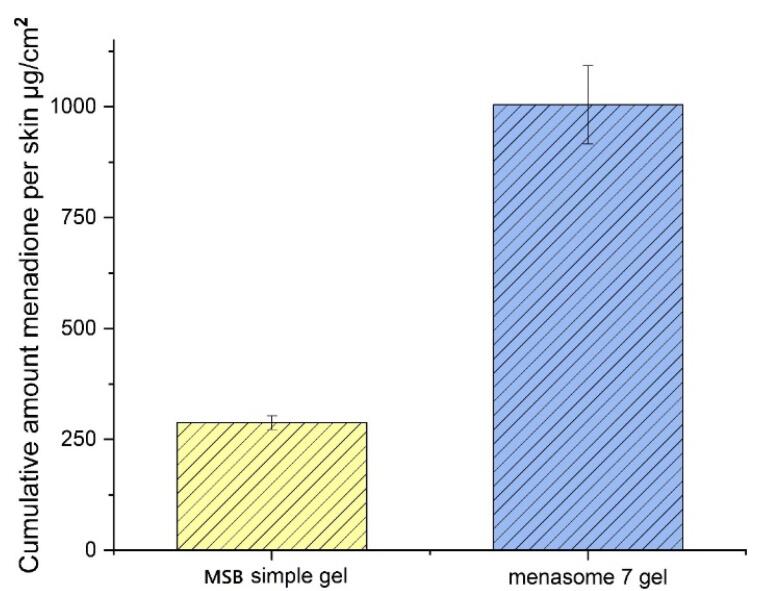
The cumulative MSB (menadione sodium bisulfite) amounts remained in rat skin

 The cumulative amounts of MSB that permeated the rat skin (Figure 5, transdermal delivery) and the amount that infiltrated the skin layers (Figure 6, dermal delivery) for Menasome 7 gel and MSB simple gel are shown in Figures 5 and 6. When compared to MSB simple gel, the Menasome 7 gel (F8) showed a greater penetration across and into the epidermal layers, indicating that it was particularly suitable for transdermal delivery (*P <*0.05). The highest MSB level identified in the compartment of the receptor for MSB simple gel (15.53 ± 1.227% or 286.200 ± 22.6 μg/cm^2^) was expressively lower than that of the Menasome 7 gel (23.827 ± 1.964% or 439.000 ± 36.19 μg/cm^2^) (*P <*0.05). Also, the level of MSB that stayed in the cutaneous for Menasome 7 gel (54.540 ± 4.790% or 1004.851 ± 88.26514884 μg/cm^2^) was significantly larger than that for the MSB simple gel (15.629 ± 1.130% or 287.948 ± 15.727 μg/cm^2^) (*P <*0.05).

 According to Chakraborti et al, enhancing drug penetration from formulations also depends on the gelling qualities of carbopol polymers.^50^ The niosome Chol concentration appeared to have an important impact on the skin’s absorption of MSBs. According to Tavano et al, Chol can alter the persistence of membrane permeation, phase behavior, and fluidity which can improve drug absorption through the skin.^30^ Topical permeability enhancers, such as Chol and its esters, are widely utilized.^51,52^ Research has demonstrated that niosome HLB value plays a significant role in determining drug cutaneous permeability.^16^ According to these results, a niosome formulation would be more effective than a conventional gel preparation for cutaneous and transdermal delivery.

###  MTT assay examination 

 Because of its ease of use, the MTT test is among the absorbance-based investigations that can be used to assess the metabolic activities of living cells. In this work, the Menasome effect on cell viability was investigated using MTT. As mentioned in section 2.9, several concentrations of Menasome, blank niosome, and free MSB solution at 1–15 µM were investigated.

**Figure 7:**
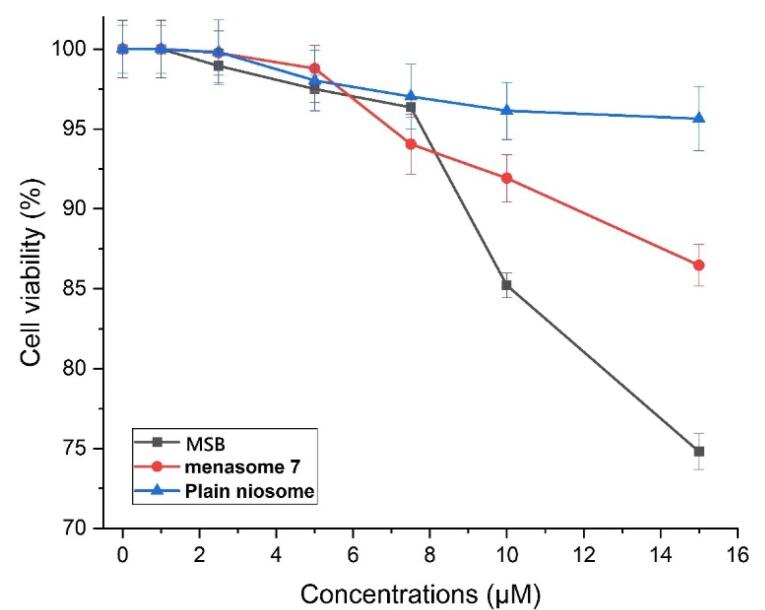
Viability of cells at diverse concentrations of MSB (menadione sodium bisulfite), Menasome 7, and plain noisome (mean ± SD, n = 5)

 HFF cells demonstrated concentration-dependent cytotoxicity following a 24-hour treatment with various concentrations of Menasome7, MSB solution, and bare niosome (Figure 7). After 24 hours of incubation with 400 µM blank niosome and Menasome 7, 96.020 ± 1.735and 86.4705 ± 1.304% of cells remained, respectively; whereas this amount was 75.24 ± 1.35% with the MSB solution. These results show that entrapping MSB in a niosome increases cell viability. This is probably because Menasomes can sustain drug release, which lowers the concentration of drug deposited close to the cell and reduces the drug’s cytotoxic effects. Furthermore, increased reactivities with proteins and cells derive from the charge density of the formulation. The prepared niosome showed a little zeta potential (-3.05). Consequently, there is less of a charge delivery around the particle and less toxicity.^38^

###  Effect of Menasome on the synthesis of melanin

**Figure 8:**
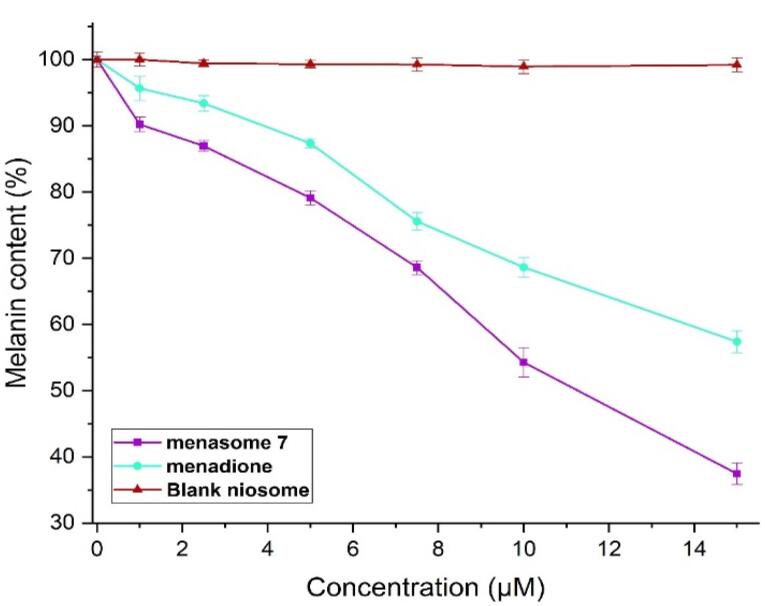
Melanin content of MSB (menadione sodium bisulfite), Menasome7, and plain niosome at various concentrations (mean ± SD, n = 5)

 The final stage of the melanogenesis process is the synthesis of melanin. A reduction in the amount of melanin indicates that Menasome 7 is inhibiting tyrosinase, which ultimately results in a reduction in the melanin content of cells. Using B16F10 cells as the cell model, the anti-melanogenic activity of the produced niosome was determined. The findings indicated that the MSB solution was less effective than the Menasome in inhibiting the formation of melanin (Figure 8). When comparing the reduction in melanin synthesis (37.426 ± 1.644%) at 15µM concentration to MSB solution (57.383 ± 1.654%), Figure 8 shows that Menasome 7 had the highest effect. MSB has been found to decrease melanin synthesis by activating the ERK (Extracellular signal-regulated kinase) pathway, a signaling system governing cellular processes. While ERK typically stimulates melanin production, MSB’s presence alters ERK activation, leading to a reduction in melanin content. Also, The ERK pathway plays a key role in anti-melanogenesis by promoting the degradation of MITF (Microphthalmia-associated transcription factor), a critical transcription factor for melanogenic enzymes like tyrosinase. When activated, ERK phosphorylates MITF, leading to its degradation and subsequent reduction in melanin production. In the B16F10 melanoma cell line, MSB (vitamin K3) has been shown to generate reactive oxygen species (ROS), which activate the ERK pathway. This activation results in the phosphorylation and degradation of MITF, thereby decreasing tyrosinase expression and melanin synthesis.^53^ The involvement of the ERK pathway in MSB’s anti-melanogenic effect is confirmed through increased phosphorylated ERK levels, reduced melanogenesis upon menadione treatment, and the reversal of these effects by ERK pathway inhibitors.^7^ The exact mechanism of how MSB influences ERK signaling in melanocytes remains unclear but likely involves multiple steps and factors. Nevertheless, MSB shows potential as a hypopigmentary agent for treating skin pigmentation disorders.^7^

###  Inhibition of L-dopa auto-oxidation assay

**Figure 9:**
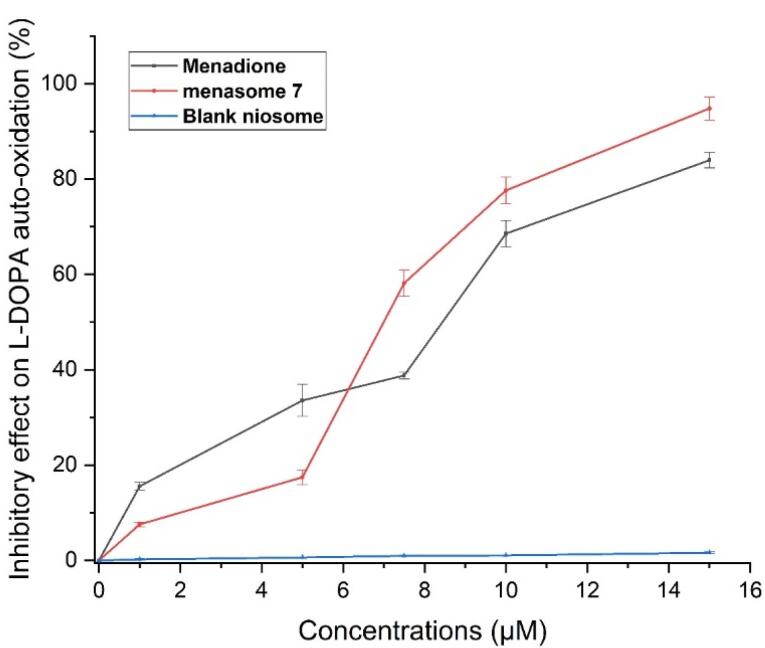
Inhibitory effect on L-dopa auto-oxidation of MSB (menadione sodium bisulfite), Menasome 7, and blank niosome (mean ± SD, n = 5)

 Effective anti-melanogenic products are crucial because controlling unattractive hyperpigmentation is the most challenging test in dermatology and cosmetics.^54^ The results of this study demonstrated that, in a concentration-dependent manner, Menasome 7 and MSB solution reduced the auto-oxidation of L-dopa. In the MSB solution, the highest inhibitory concentration was 83.953 ± 1.629% at 15 µM, as shown in Figure 9. On the other hand, Figure 9 shows that an increase in Menasome concentration corresponds with an increase in inhibition (94.806 ± 2.411%). This profile shows that Menasome 7 activity is significantly inhibited at dosages ranging from 1 to 5 µM. The release curve of vitamin K from hydrogel (Figure 1) explains this action. Compared to pure MSB (positive control) with an IC50 value of 9.187 ± 0.48 µM (*P* < 0.05), Menasome 7 had a considerable inhibitory impact against L-DOPA auto-oxidation (IC50 = 7.13 ± 0.279 µM). MSB performs as an antioxidant by diminishing the detrimental ROS and free radicals produced throughout the auto-oxidation of L-DOPA. MSB aids in safeguarding L-DOPA from oxidative destruction by diminishing ROS amounts, hence potentially amplifying its therapeutic efficacy.^55,56^ The outcomes suggest that adding MSB to the niosome could enhance the inhibitory auto-oxidation process, which could be useful for usage as a skin-lightening cosmetic.

###  Irritation test on the rat skin

 In addition to administering the drug, the pharmaceutical delivery system should not significantly irritate the patient or have harmful or immunogenic effects. To be considered an appropriate preparation, the Menasome 7 preparation must not cause skin hypersensitivity because it is intended for topical application.^57^ As a matter of fact, one of the most frequent adverse effects of dermal topical therapies is skin irritation, and it is directly related to the concentration of the active substance. Therefore, modifying the preparation’s chemical release could reduce its adverse effects by increasing intra-follicular penetration and declining cutaneous uptake.^57^ The impact of different preparations of MSB on the measurement of skin irritation (erythema and edema) is displayed in Table 2. Draize, Woodward, and Calvery have classified compounds with grade levels of 2 or lower as negative (no skin irritation). The edema and erythema rating of 0.333 for the Menasome 7 gel preparation was significantly lower than the value recorded for formalin and other MSB preparations (Table 2). This shows no irritation and improves the tolerance and dermal suitability of the Menasome 7 formulation.

**Table 2:** Cutaneous sensitivity values after local application

**Rat No.**	**Control**		Menasome 7 gel*		**MSB plain gel**		**Blank niosomal gel**		**Formalin**	
	**Erythema**	**Edema**	**Erythema**	**Edema**	**Erythema**	**Edema**	**Erythema**	**Edema**	**Erythema**	**Edema**
1	0	0	0	0	0	0	1	0	4	3
2	0	0	0	0	1	0	0	0	3	4
3	0	0	0	0	1	1	1	1	4	3
4	0	0	0	1	1	0	1	0	4	4
5	0	0	0	0	0	1	1	0	3	3
6	0	0	1	0	2	2	0	0	3	3
Score	0		0.333		1.5		0.833		6.833	

* Significant compared with formalin MSB plain gel (*P <*0.05).

 This indicates the lack of sensitivity, hence improving the tolerance and compatibility of the Menasome formulation with the dermis. Further investigations have also observed comparable results in experiments that examined niosomal preparations of nortriptyline,^58^ terbinafine,^59^ kojic acid,^33^ and venlafaxine.^16^

## Conclusion

 The hydrophilic drug MSB has been successfully added to niosomes made of Chol and nonionic surfactants. MSB in niosomes was in an amorphous state and did not interact chemically with the other niosome elements because of solid-state testing. The average encapsulation efficiency, particle size, PDI, and zeta potential of the adjusted niosome topical preparation were 83.616 ± 2.550%, 298.133 ± 8.823nm, 0.304 ± 0.006, and -3.056 ± 0.797 mV, respectively. Moreover, due to the developed Menasome’s higher percentage of cell survival approximately 85%, the MTT investigation discovered no cell toxicity in HFFs. Besides, the applied Menasome 7 gel constituents were presented to be non-irritant in a dermal irritation test. When compared to free MSB sodium bisulfite and blank niosome, the anti-hyperpigmentation activity of niosome loaded with MSB showed the strongest effect, including reduction of melanin production and L-Dopa auto-oxidation inhibitory actions. This work has demonstrated the potential application of MSB-loaded green technology niosomes as preparation of anti-hyperpigmentation for use in cosmetic and pharmaceutical products.

## Competing Interests

 No conflicts of interest were reported by the authors.

## Data Availability Statement

 Data will be available upon request.

## Ethical Approval

 The ethical review board for animal studies at Mazandaran University of Medical Sciences approved the animal experiments, and the ethical code was IR.MAZUMS.REC.1400.10517.
